# Ambient Levels of Air Pollution Induce Goblet-Cell Hyperplasia in Human Conjunctival Epithelium

**DOI:** 10.1289/ehp.10363

**Published:** 2007-09-14

**Authors:** Priscila Novaes, Paulo Hilário do Nascimento Saldiva, Newton Kara-José, Mariângela Macchione, Monique Matsuda, Lourdes Racca, Alejandro Berra

**Affiliations:** 1 Laboratório de Investigação em Oftalmologia, Faculdade de Medicina, Universidade de São Paulo, São Paulo, Brasil; 2 Laboratório de Poluição Atmosférica Experimental, Faculdade de Medicina, Universidade de São Paulo, São Paulo, Brasil; 3 Laboratorio de Investigaciones Oculares, Facultad de Medicina, Universidad de Buenos Aires, Buenos Aires, Argentina

**Keywords:** air pollutants, conjunctiva, environmental, goblet-cell, impression cytology, nitrogen dioxide

## Abstract

**Background:**

Ocular mucosa is exposed constantly to the external environment, and chronic exposure to air pollution may affect the ocular surface.

**Objective:**

We assessed the effect of air pollution on the ocular surface by combining determinations of individual exposure and conjunctival impression cytology.

**Methods:**

A panel study was conducted with 29 volunteers recruited in two locations with different pollution levels: São Paulo (*n* = 13) and Divinolândia (*n* = 16). We assessed mean individual levels of nitrogen dioxide (NO_2_) exposure for 7 days, using a passive sampler. Impression cytology samples were obtained from inferior tarsal conjunctiva. Comparisons between the two groups in terms of NO_2_ exposure and goblet-cell counts were performed using the Student *t*-test. Correlations between goblet-cells counts and corresponding individual NO_2_ exposure levels were determined using Spearman’s correlation.

**Results:**

Individuals living in São Paulo received a significantly (*p* = 0.005) higher dose of NO_2_ (mean 32.47; SD 9.83) than those living in Divinolândia (mean 19.33; SD 5.24). There was a steady increase in goblet-cell counts, proportional to NO_2_ exposure (Spearman’s correlation = 0.566, *p* = 0.001), with a dose–response pattern.

**Conclusions:**

A positive and significant association between exposure to air pollution and goblet-cell hyperplasia in human conjunctiva was detected. The combination of simple measurements of exposure and impression cytology was an effective and noninvasive approach for characterizing human response to ambient levels of air pollution.

Acute adverse health effects of ambient levels of air pollution have been demonstrated in humans, mostly in terms of respiratory and cardiovascular events (Barnett et al. 2006; Ito et al. 2005; Medina-Ramón et al. 2006; Pope et al. 2002; Samoli et al. 2003; Souza et al. 1998).

Ocular mucosa is exposed constantly to the external environment. Indeed individuals living in areas with high concentrations of pollutants frequently report ocular symptoms (Saxena et al. 2003; Versura et al. 1999), and previous studies have detected tear film abnormalities and subclinical changes of the ocular surface in individuals who lived in cities with high levels of air pollution (Gupta et al. 2002; Saxena et al. 2003). In such scenario, changes in ocular mucosa may indicate potential damage to the eyes and represent a convenient biomarker of the adverse health effects induced by air pollution if objective estimators of ocular surface changes in individuals living in urban areas are proportional to the degree of exposure. In the present study we explored this concept by conducting a panel study combining determinations of individual exposure to air pollution, expressed in terms of nitrogen dioxide (NO_2_) and measuring ocular surface changes by impression cytology.

## Materials and Methods

### Study population

The study involved 29 volunteers, who were recruited in two locations, with different pollution levels: São Paulo (*n* = 13) and Divinolândia (*n* = 16). São Paulo is the largest city in Latin America, with high levels of air pollution, mainly as a result of traffic emissions. Divinolândia is a small city in the countryside of the state of São Paulo, where half the population lives in rural settings and where there is no significant industrial activity. The volunteers were recruited among the employees of two public hospitals: at the General Clinics Hospital of the University of São Paulo Medical School and the Regional Hospital in Divinolândia. The hospital in São Paulo is located downtown at an intersection of broad avenues with heavy traffic, whereas in Divinolândia the hospital is located in a relatively isolated spot surrounded by farms. The research protocol was approved by the ethics committees of both institutions, and all subjects gave their informed consent before enrollment in the study. The following inclusion criteria were adopted: *a*) to be part of the fixed staff of the hospitals, *b*) to have been living in the study area for at least 5 years, and *c*) to accept participating in the study after reading and signing an informed consent form. The following parameters were adopted to exclude volunteers: *a*) smoking, *b*) possibility of traveling to other areas during the monitoring period (7 days), *c*) contact with chemical solutions such as organic solvents, sodium hypochloride, and formalin, and *d*) individuals with history of use of contact lenses, ophthalmic surgery, and preexisting ophthalmic conditions. All volunteers used gas cooking and no houses had home heating.

### Exposure assessment

NO_2_ was used as an indicator of exposure to air pollution. We employed a passive sampler that included a cellulose filter (Energetica, Rio de Janeiro, Brazil) impregnated with an absorbant solution—trietanolamine 2%, 0.05% *o*-methoxyphenol, and 0.025% sodium metabisulfite (Lodge 1989), which was enclosed within a small plastic tube with one of its extremities open to ambient air. The nitrite produced during sampling was determined colorimetrically by reacting the exposed absorbing reagent with sulfanilamide and 8-anilino-1-naphthalene-sulfonic acid (ANSA) at a wavelength of 550 nm.

**Figure 1:**
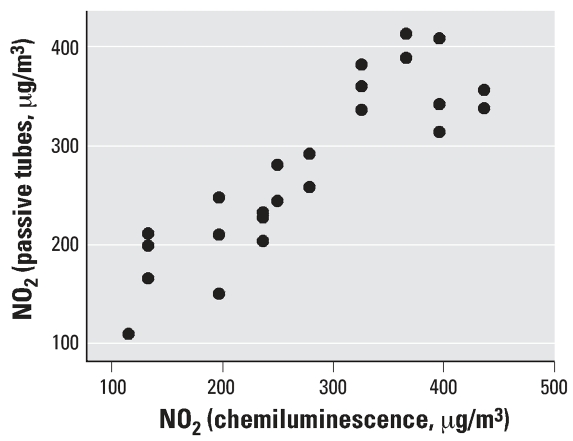
Graphic representation of simultaneous determinations of accumulated ambient concentrations of NO_2_, measured by the passive sampler and corresponding values determined by the Sanitation Agency of the São Paulo (CETESB 2007).

Before the study we evaluated the efficiency and sensitivity of this measuring system by placing the filters near an automatic chemiluminescence monitor of the State Sanitation Agency of São Paulo (CETESB 2007). Our method has an accuracy of 98.6% and a precision of 80%. The lower detection limit is an accumulated exposure of 100 μg/m^3^_,_ and no evidence of saturation was observed up to 450 μg/m^3^ of accumulated exposure (defined as the average concentration of 24 hr of NO_2_ exposure per number of days). The accumulated NO_2_ concentration obtained by the two methods was compared across a time window ranging from 3 to 10 days. A good agreement between the two methods was observed, as depicted in Figure 1.

Passive samplers were fixed in the pocket of the studied individuals, who were instructed to keep them at all times during their daily activities and by the bedside during the night for 7 days.

We used a cumulative measure of 1 week of NO_2_ exposure and divided such measure by 7, which gave us a mean individual level of exposure that was compared with goblet-cell counts.

### Impression cytology of conjunctival mucosa

Impression cytology was used to obtain samples from the superficial epithelial cell layers of inferior tarsal conjunctiva. Impression cytology samples were collected after the 7-day monitoring of NO_2_. All samples were collected in the morning (0900–1100 hours).

Semicircular filters approximately 15 mm diameter in size (cellulose ester filter 22-μm pore; Millipore Corp., Bedford, MA, USA) were applied to the inferior tarsal conjunctiva after instillation of one drop of topical anesthetic (tetracaine) in each eye, and the excess fluid was wiped away. The paper fragments were applied for approximately 10 sec, and after gentle pressure with the blunt end of the forceps, the fragments were peeled off and immediately immersed in tubes containing absolute ethanol. After fixation, specimens were rehydrated in 70% ethyl alcohol, then placed successively in periodic acid–Schiff reagent, sodium metabisulfite, Gill’s hematoxylin and Scott’s tap water. Specimens were then rinsed with 95% alcohol and absolute alcohol. Xylene was used to make the filter paper transparent. Before mounting, the filter paper was placed with epithelial cells facing up. Slides were examined by light microscopy and goblet cells were counted in 10 high-power fields (HPF) with a 400× magnification (Singh et al. 2005). The same investigator (A.B.) performed the goblet-cell counts and was blinded to the place of origin of the samples.

### Statistical analysis

Comparisons of NO_2_ exposure and goblet-cell counts between the two groups (São Paulo and Divinolândia) were performed using the Student *t*-test, with corrections for inequality of variances. Correlations between goblet-cell counts and corresponding individual NO_2_ exposure level were determined using Spearman’s correlation. The effect of air pollutants on goblet-cell counts was also measured by analysis of variance (ANOVA) and Bonferroni’s post hoc test. The level of significance was set at 5%. Goblet-cell counts were performed in Argentina, and NO_2_ data were simultaneously computed in São Paulo. The two data sets were combined only after the analysis of the samples was concluded.

## Results

There were no significant differences among the groups in São Paulo (SP) and Divinolândia (D) regarding age (SP: mean 37.15, SD 8.2; D: mean 32.13, SD 9.2; *p* = 0.136); sex (SP: female, 85%, male, 15%; D: female, 94%, male, 6%); and passive smoking (SP, 37.5%; D, 38.5%).

**Figure 2:**
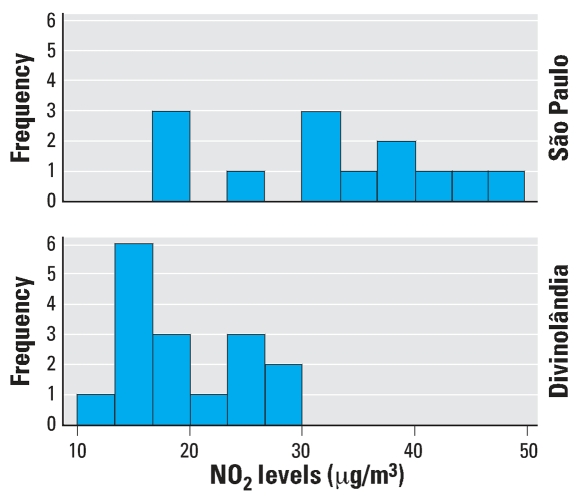
Individual NO_2_ exposure levels in each group.

Individuals living in São Paulo received a significantly (*p* = 0.005) higher dose of NO_2_ (mean 32.47, SD 9.83) than those in Divinolândia (mean 19.33, SD 5.24), as expected based on the characteristics of each city (Figure 2).

Mean (and corresponding SD) number of goblet cells per 10 HPF was 243.37 ± 132.67 in Divinolândia and 325.80 ± 147.90 in São Paulo. The difference between the two groups was not significant (*p* = 0.102, Student *t*-test). The difference between goblet-cell counts was not statistically significant in our sample because of the presence in the Divinolândia group of an outlier with a goblet-cell count of about 600 cells in 10 HPF. If this outlier were excluded, individual differences would be significant (*p* = 0.029, Student *t*-test).

**Figure 3:**
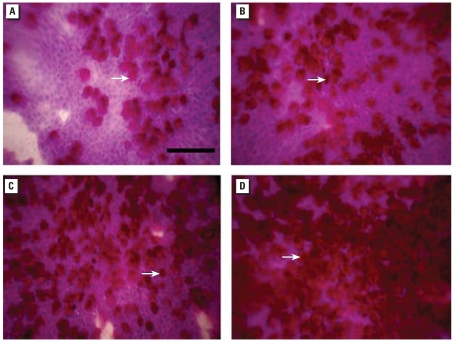
Impression cytology photographs of representative samples. The arrows indicate goblet cells. Corresponding goblet-cell counts and NO_2_ exposure levels are: *(A*) 194 cells × 10HPF NO_2_ 13.10 μg/m^3^; (*B*) 357 goblet cells × 10HPF NO_2_ 18.04 μg/m^3^; (*C*) 409 goblet cells × 10HPF NO_2_ 26.89 μg/m^3^; (*D*) 575 goblet cells × 10HPF NO_2_ 45.31 μg/m^3^. Scale bar = 100 μm.

We classified subjects’ exposures to NO_2_ into four categories on the basis of quartiles of NO_2_ levels: Q1, < 17 μg/m^3^; Q2, 18–21 μg/m^3^; Q3, 22–33 μg/m^3^; and Q4, > 33 μg/m^3^, regardless of the city of residence. The mean counts of goblet cells, distributed by quartiles of NO_2_ were Q1, 186 cells; Q2, 253 cells; Q3, 312 cells; and Q4, 402 cells. There was a 216% increase in the number of goblet cells from the first to the fourth quartile. Figure 3 shows the microscopic aspect of impression cytology samples, and a marked increase in goblet-cell hyperplasia can be observed in subjects exposed to higher levels of NO_2_.

**Figure 4:**
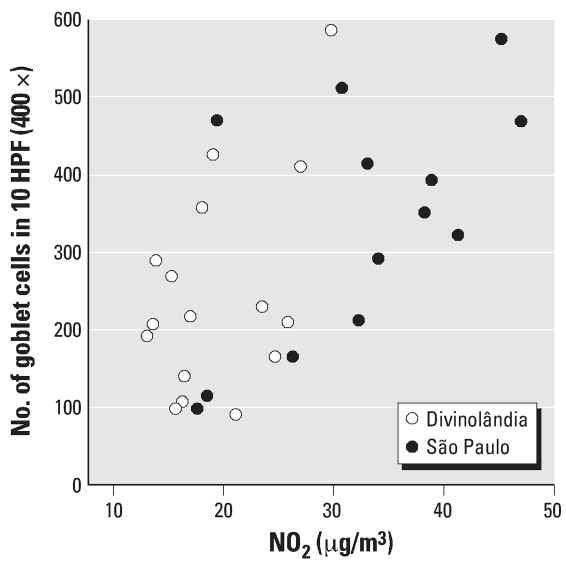
Individual cell counts plotted as a function of NO_2_ exposure levels, indicated by study location.

Goblet-cell counts increased proportionately to NO_2_ exposure in a dose–response pattern despite the variability observed (Figure 4). Indeed, Spearman’s correlation between NO_2_ and goblet cells per 10 HPF was positive (0.566) and significant (*p* = 0.001). Using ANOVA to measure the effect of air pollutants on goblet-cell counts, we detected a significant difference among groups (*p* = 0.036). The Bonferroni post hoc test indicated that goblet-cell counts of the first and fourth quartiles of NO_2_ exposure were significantly different.

## Discussion

Goblet-cell hyperplasia is a stereotyped response of mucosal surfaces when chronically exposed to air pollution, as demonstrated in rodents (Pires-Neto et al. 2006; Saldiva et al. 1992) and humans (Souza et al. 1998). In this present study, we report that conjunctival mucosa follows this same path. In addition, we demonstrated a positive and significant correlation between gradients of exposure and intensity of goblet-cell hyperplasia.

Conjunctival goblet cells are slow cycling cells that may proliferate in response to chronic inflammatory stimuli (Dartt 2004; Pellegrini et al. 1999; Wei et al. 1995). Goblet-cell hyperplasia is detected in chronic allergic eye disease such as vernal keratocon-junctivitis, atopic keratoconjunctivitis, and giant papillary conjunctivitis (Mc Dermott et al. 2005). Studies have shown that chronic exposure to ambient air pollution is associated with subclinical changes of the ocular surface and the tear film (Gupta et al. 2002; Saxena et al. 2003), but goblet-cell hyperplasia related to NO_2_ has not been documented previously. There is no accurate characterization of goblet-cell turnover in conjunctival mucosa; however, there is substantial information on mucous hyperplasia of the airways resulting from exposure to sulfur dioxide (SO_2_) and ozone across a wide range of exposure protocols (Fanucchi et al. 2006; Harkema et al. 1999; Saldiva et al. 1992; Seltzer et al. 1984; White et al. 1986). Generally, mucous hyperplasia occurs in the respiratory epithelium after weeks or months and is considered a marker of airway remodeling induced by persistent injurious stimuli. The observed mucous hyperplasia probably reflects exposure to air pollution that occurred within a much larger time window than the 1-week assessment that was conducted.

We considered NO_2_ a proxy estimator of exposure to air pollution, not the single causative agent. The passive sampler we employed for the determination of NO_2_ exposure was simple, did not restrict daily activities and exhibited a good correlation with the state monitoring system (Figure 1). In addition, previous studies our group conducted in the last decade observed consistent and significant correlations between NO_2_ and other primary pollutants such as PM_10_ (particulate matter with an aerodynamic diameter ≤ 10 μm), carbon monoxide, and SO_2_ (Braga et al. 2001; Conceição et al. 2001; de Paula Santos et al. 2005; Farhat et al. 2005; Lin et al. 2003, 1999; Martins et al. 2002; Pereira et al. 1998; Saldiva et al. 1995). Thus, NO_2_ should be considered, within the context of the current study, a marker of combustion-derived pollutants produced by vehicular emissions and other sources such as biomass burning.

In the present study the cumulative measure of 1 week of NO_2_ exposure was considered a representative measure of chronic exposure, and the study was conducted simultaneously in both cities to minimize climatic influence.

The range of exposure to NO_2_ experienced by our study population was between 10 and 50 μg/m^3^ (24-hr mean of 1-week exposure). Such levels are within those observed in several locations throughout the world, indicating that the individuals evaluated in this study were not exposed to extremely high levels of air pollution. Figure 4 suggests that the relationship between NO_2_ and goblet-cell hyperplasia is linear and without a threshold or plateau. Although our sample was small, it is tempting to speculate that exposure to low levels of air pollution may induce subclinical damage, which ultimately leads to an adaptive response of the conjunctival epithelium. Our findings reinforce the concept that populations exposed to ambient levels of air pollution are subjected to possible chronic injury, which includes, as discussed in the present study, the ocular surface.

Figure 4 shows a substantial degree of variability in the responses to similar levels of air pollution. This finding suggests that some individuals have greater susceptibility to airborne toxic agents. Our study was not designed to explore this point, but it would be interesting to explore in further studies whether ocular mucosa may be a convenient scenario to study the determinants—genetic or epigenetic—of the susceptibility of mucosal surfaces to air pollution.

In the present study we observed a positive and significant association between exposure to air pollution and goblet-cell hyperplasia in human conjunctiva. The combination of simple measurements of exposure and impression cytology was shown to be an effective and non-invasive approach to characterize human response to ambient levels of air pollution.
